# AcrB, AcrD, and MdtABC Multidrug Efflux Systems Are Involved in Enterobactin Export in *Escherichia coli*

**DOI:** 10.1371/journal.pone.0108642

**Published:** 2014-09-26

**Authors:** Tsukasa Horiyama, Kunihiko Nishino

**Affiliations:** 1 Graduate School of Pharmaceutical Sciences, Osaka University, Osaka, Japan; 2 Laboratory of Microbiology and Infectious Diseases, Institute of Scientific and Industrial Research, Osaka University, Osaka, Japan; Institut National de la Recherche Agronomique, France

## Abstract

*Escherichia coli* produces the iron-chelating compound enterobactin to enable growth under iron-limiting conditions. After biosynthesis, enterobactin is released from the cell. However, the enterobactin export system is not fully understood. Previous studies have suggested that the outer membrane channel TolC is involved in enterobactin export. There are several multidrug efflux transporters belonging to resistance-nodulation-cell division (RND) family that require interaction with TolC to function. Therefore, several RND transporters may be responsible for enterobactin export. In this study, we investigated whether RND transporters are involved in enterobactin export using deletion mutants of multidrug transporters in *E. coli*. Single deletions of *acrB*, *acrD*, *mdtABC*, *acrEF*, or *mdtEF* did not affect the ability of *E. coli* to excrete enterobactin, whereas deletion of *tolC* did affect enterobactin export. We found that multiple deletion of *acrB*, *acrD*, and *mdtABC* resulted in a significant decrease in enterobactin export and that plasmids carrying the *acrAB*, *acrD*, or *mdtABC* genes restored the decrease in enterobactin export exhibited by the Δ*acrB acrD mdtABC* mutant. These results indicate that AcrB, AcrD, and MdtABC are required for the secretion of enterobactin.

## Introduction

Multidrug efflux transporters cause serious problems in cancer chemotherapy and in the treatment of bacterial infections. In gram-negative bacteria, transporters belonging to the resistance-nodulation-cell division (RND) family are particularly effective in generating resistance because they form a tripartite complex with periplasmic proteins and an outer membrane protein channel. The RND transporters have wide substrate specificity [1]. The AcrAB–TolC system is composed of the RND transporter AcrB, membrane fusion protein AcrA, and multifunctional outer membrane channel TolC. It has been suggested that the AcrAB–TolC multidrug efflux system is capable of capturing substrates in the periplasm rather than in the membrane or the cytoplasm [2]. This claim is supported by high-resolution structures of AcrB, in which access pathways from the periplasm, but not from the cytoplasm, have been identified [3], [4].

TolC plays an important role in the excretion of a wide range of molecules in *E. coli*, including antibiotics [5]–[7], bile salts [8]–[10], organic solvents [11], several antibacterial peptides such as colicin V [12], [13] and microcin J25 [14] and a large protein toxin, α-hemolysin [15], [16]. TolC interacts with a variety of inner membrane transporters and enables *E. coli* to expel structurally diverse molecules. In *E. coli*, AcrB, AcrD, AcrEF, MdtABC, and MdtEF belong to the RND transporters and require TolC to function [7], [17].

Iron is an essential element for many biological processes, such as amino acid and nucleotide synthesis, electron transport, and peroxide reduction [18]. Many bacteria excrete iron-chelating compounds called siderophores to grow under iron-limited conditions. *E. coli* can produce the catecholate siderophore enterobactin (also called enterochelin), which is a cyclic triester of 2, 3-dihydroxybenzoylserine (DHBS) [19], [20]. Enterobactin is synthesized from chorismate in the cytoplasm [21] and exported from the cell. Extracellular iron-loaded enterobactin is taken up via the outer membrane receptor FepA and translocated to the periplasm [22], [23]. Fe-enterobactin is chaperoned by FepB to the ATPase-dependent transporter FepDGC and shuttled to the cytoplasm. In the cytoplasm, enterobactin is degraded by Fes esterases to release iron [24]–[27].

The systems responsible for enterobactin synthesis and uptake are well characterized, as presented above. In contrast, the enterobactin export system is not fully understood. Previously, enterobactin export across the cytoplasmic membrane was shown to be dependent on the major-facilitator transporter EntS (the *ybdA* gene product) [28]. EntS has also been shown to be responsible for enterobactin export in *Salmonella enterica* serovar Typhimurium [29]. Bleuel *et al*. showed that the outer membrane channel TolC is involved in enterobactin export from the periplasm to the culture medium [30]; however, the RND transporters required for enterobactin export have not yet been identified. The RND transporters have wide substrate specificity and require TolC for their function. Therefore, it is possible that the RND transporters are responsible for enterobactin export. In this study, we investigated whether RND transporters are involved in enterobactin export using several deletion mutants of RND transporter genes and high-performance liquid chromatography analysis.

## Materials and Methods

### Bacterial strains and growth conditions

Bacterial strains and plasmids used in this study are listed in Table 1. The *E. coli* strains used in this study are derived from the wild-type strain MG1655 [31]. For production and detection of enterobactin, strains were grown at 37°C for 13 h with shaking in iron-restricted T medium containing the following per liter: 5.8 g NaCl, 3.7 g KCl, 0.113 g CaCl_2_, 0.1 g MgCl·6H_2_O, 1.1 g NH_4_Cl, 0.272 g KH_2_PO_4_, 0.142 g Na_2_SO_4_, and 12.2 g Tris. The pH was adjusted to 7.4 with 1 N HCl, and medium was supplemented with 33.4 ml deferrated casamino acids, 0.4% (wt/vol) glucose, 0.005% (wt/vol) nicotinic acid, 1% (wt/vol) thiamine before inoculation, and 150 µM 2,2′-dipyridyl. Deferrated casamino acids were prepared using the following methods. Twenty-five ml of 10% (wt/vol) casamino acids were mixed with 0.75 g hydroxyquinoline and 25 ml chloroform. Thereafter, the supernatant was again mixed with 25 ml chloroform and its supernatant was collected as deferrated casamino acids. Aliquots of iron-restricted T medium were inoculated with 0.1% (v/v) of overnight cultures of strains grown in Luria-Bertani (LB) broth (1% tryptone, 0.5% yeast extract, 0.5% NaCl) [28], [30], [32]. Ampicillin was added to the growth medium at the final concentration of 100 mg/L for plasmid maintenance.

**Table 1:** Strains and plasmids used in this study.

Strain or Plasmid	Original name	Characteristics	Source or references
Strains as in text			
Wild-type	MG1655	*Escherichia coli* wild type	31
Δ*acrB*	NKE96	Δ*acrB*	36
Δ*acrD*	NKE94	Δ*acrD*::Cm^R^	37
Δ*mdtABC*	NKE133	Δ*mdtABC*::Km^R^	37
Δ*acrEF*	NKE129	Δ*acrEF*::Km^R^	This study
Δ*mdtEF*	NKE138	Δ*mdtEF*::Km^R^	34
Δ*tolC*	NKE95	Δ*tolC*::Cm^R^	35
Δ*entS*	NKE869	Δ*entS*	This study
Δ*acrB acrD*	NKE126	Δ*acrB* Δ*acrD*	37
Δ*acrB mdtABC*	NKE141	Δ*acrB* Δ*mdtABC*::Km^R^	37
Δ*acrD mdtABC*	NKE1288	Δ*mdtABC*::Km^R^ Δ*acrD*::Cm^R^	This study
Δ*acrB acrD mdtABC*	NKE1317	Δ*acrB* Δ*acrD* Δ*mdtABC*	37
Δ*acrB acrD mdtABC mdtEF*	NKE1327	Δ*acrB* Δ*acrD* Δ*mdtABC* Δ*mdtEF*	This study
Δ*acrB acrD mdtABC mdtEF acrEF*	NKE1329	Δ*acrB* Δ*acrD* Δ*mdtABC* Δ*mdtEF* Δ*acrEF*::Km^R^	This study
Δ*acrB acrD mdtABC*/vector	NKE1575	Δ*acrB* Δ*acrD* Δ*mdtABC*/pTrc99A	This study
Δ*acrB acrD mdtABC*/p*acrAB*	NKE1576	Δ*acrB* Δ*acrD* Δ*mdtABC*/p*acrAB*	This study
Δ*acrB acrD mdtABC*/p*acrD*	NKE1578	Δ*acrB* Δ*acrD* Δ*mdtABC*/p*acrD*	This study
Δ*acrB acrD mdtABC*/p*mdtABC*	NKE1583	Δ*acrB* Δ*acrD* Δ*mdtABC*/p*mdtABC*	This study
Vector			
pKD4		rep_R6Kγ[ρ]_ Ap^R^ FRT Km^R^ FRT	33
pCP20		rep_pSC101_^ts^Ap^R^ Cm^R^ *cI857*λP_R_ *flp*	33
pTrc99A		Vector, Ap^R^	Amercham Pharmacia Biotech
Plasmids			
p*acrAB*		*acrAB* genes cloned into pTrc99A, Ap^R^	This study
p*acrD*		*acrD* genes cloned into pTrc99A, Ap^R^	This study
p*mdtABC*		*mdtABC* genes cloned into pTrc99A, Ap^R^	This study

### Construction of gene deletion mutants

To construct the drug efflux mutants, gene disruption was performed as described by Datsenko and Wanner [33]. The mutants Δ*tolC*, Δ*acrB*, Δ*acrD*, Δ*mdtABC*, Δ*mdtEF*, Δ*acrB acrD*, Δ*acrB mdtABC*, and Δ*acrB acrD mdtABC* were constructed as previously described [34]–[37]. The oligonucleotide primers *acrE*-P1 (TTGGGTAAATAACGCGCTTTTGGTTTTTTGAGGAATAGTAGTGTAGGCTGGAGCTGCTTC) and *acrF*-P2 (AAATAATAAAGGCACCCGAAAGCGCCTTTATGTTTCTGATCATATGAATATCCTCCTTAG) were used to construct the Δ*acrEF* mutant. The kanamycin resistance gene *aph*, flanked by Flp recognition sites, was amplified by polymerase chain reaction (PCR) using the primers listed above. The resulting PCR products were used to transform the recipient MG1655 strain harboring the plasmid pKD46, which expresses lambda Red recombinase. The chromosomal structures of the mutated loci were verified by PCR using the primers *acrE*-F (GTTAAATAAATAATATATATTATTTACCTA) and *acrF*-R (CGTGAGCACAGCCCGCCAGCAATGCGGTGA), and K-1 (CAGTCATAGCCGAATAGCCT) and K-2 (CGGTGCCCTGAATGAACTGC). To construct Δ*acrD mdtABC*, Δ*acrB acrD mdtABC mdtEF*, and Δ*acrB acrD mdtABC mdtEF acrEF* mutants, the individual deletions were transferred to a fresh isolate of MG1655 by P1 transduction. The *cat* or *aph* genes were eliminated using plasmid pCP20, as previously described [33]. To construct the Δ*entS* mutant, precise in-frame deletions were generated using crossover PCR. The oligonucleotide primers *entS*-No (CGCGGATCCAAAGGCAACAATCAATGAGGC) and *entS*-Ni (CACGCAATAACCTTCACACTCCAAATTTATAACCATTACAATGCCTTGCCATC), plus *entS*-Ci (GTTATAAATTTGGAGTGTGAAGGTTATTGCGTGTAATGCTTAAAACAGCGCCTTAAGCC), and *entS*-Co (CGCGTCGACGACGACAAAACGTGGCAGTC) were used. Then the fragment containing the deletion was cloned into the *Bam*HI site of vector pKO3, after which the deletion was introduced into the chromosome by gene replacement, as previously described [38].

### Construction of plasmids

The *acrAB* and *acrD* genes were subcloned from pHSG*acrAB* and pHSG*acrD* into vector pTrc99A using *Bam*HI and *Hind*III, and *Hind*III and *Sac*I, respectively [7]. PCR with PrimeSTAR GXL DNA polymerase (TaKaRa Bio Inc., Otsu, Japan) and the primers *mdtABC*-*Eco*RI (CGCGAATTCAAGAAATCCTTTCTTAGAGAA) and *mdtABC*-*Kpn*I (CGCGGTACCTTACTCGGTTACCGTTTGTTT) was used to amplify *mdtABC* from the chromosomal DNA of *E. coli* MG1655. This process introduced the restriction enzyme recognition site present in the multicloning region of the pTrc99A vector. The DNA fragments were digested with restriction enzymes and then ligated into the multicloning region of pTrc99A.

### Extraction of enterobactin

Enterobactin was prepared from supernatants separated from approximately 10^9^ cells of each strain. Supernatants of 13 h cultures were acidified with 50 µl 12 N HCl per 10 ml and extracted twice with 5 ml ethyl acetate. Thereafter, the ethyl acetate phase was concentrated by evaporation (Buchi, Postfach, Switzerland). Dried residues were resuspended in 500 µl methanol and analyzed by reverse-phase (RP) high-performance liquid chromatography (HPLC).

### HPLC analysis

RP HPLC analysis was performed on a Symmetry C18 Column (C_18_, 4.6×250 mm, 5 µm: Waters Corp, Milford, MA, USA) using a LaChrom Elite instrument (Hitachi, Tokyo, Japan) containing a D-2000 interface, a L-2130 pump, and a L-2400 UV detector. The mobile phase consisted of 0.075% (vol/vol) trifluoroacetic acid in H_2_O (pH 2) and acetonitrile. The flow rate was adjusted to 1 ml min^−1^, and 20 µl of each supernatant extraction was injected and separated as described by the manufacture of the standard, with monitoring at 250 nm. Peaks were identified using HPLC-grade enterobactin standards (EMC Microcollections GmbH, Tubingen, Germany). The amount of enterobactin exported was calculated from peak areas and normalized to that of the wild-type using HITACHI Model D-2000 Elite HPLC System Manager software.

## Results

### TolC is required for enterobactin export

**Figure 1:**
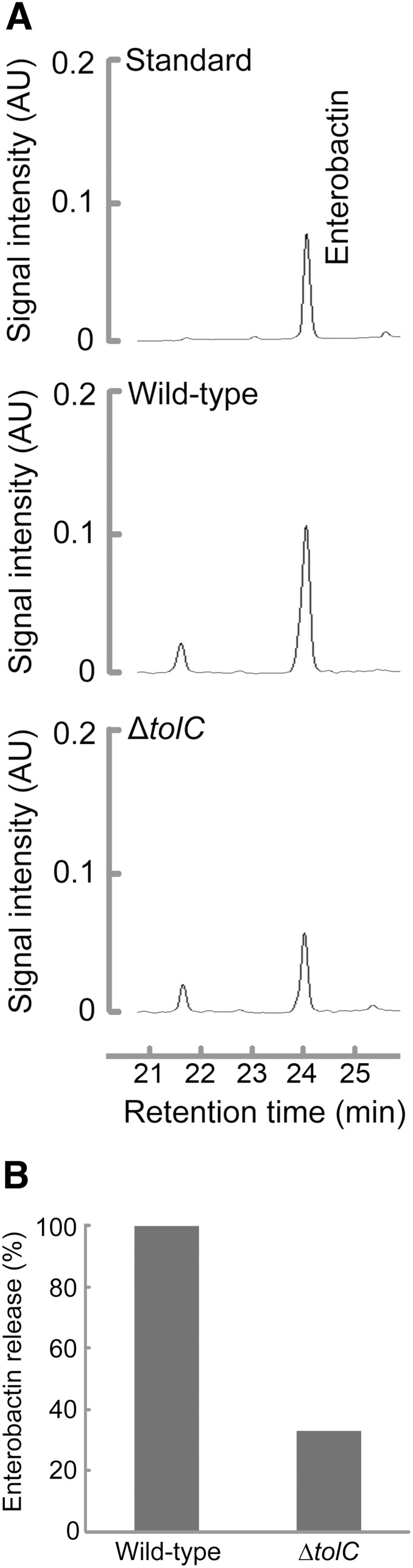
RP HPLC analysis of enterobactin release from the wild-type strain and the Δ*tolC* mutant. (A) Chromatograms of enterobactin prepared from the supernatants of the wild-type strain and the Δ*tolC* mutant. Peaks were identified using HPLC-grade standards. (B) The amount of enterobactin exported by Δ*tolC* mutant relative to that exported by the wild-type was calculated using the peak areas. The peak area corresponding to enterobactin released from the wild-type strain was defined as 100%.

Bleuel *et al*. have shown that TolC is involved in the efflux of enterobactin across the outer membrane of *E. coli* [30]. We performed HPLC analysis to confirm whether a strain with a deletion of *tolC* gene differs from the wild-type in its ability to release enterobactin. The *E. coli* wild-type and Δ*tolC* strains were grown for 13 h and then enterobactin was extracted from supernatants of these cultures for RP HPLC analysis. Deletion of *tolC* resulted in a decrease in the export of enterobactin compared with the wild-type strain (Figure 1A). When the peak area of enterobactin released from the wild-type strain was defined as 100%, enterobactin release from Δ*tolC* was decreased to 47% (Figure 1B).

### Effect of deletion of individual TolC-dependent drug efflux genes and *entS* on enterobactin release

**Figure 2:**
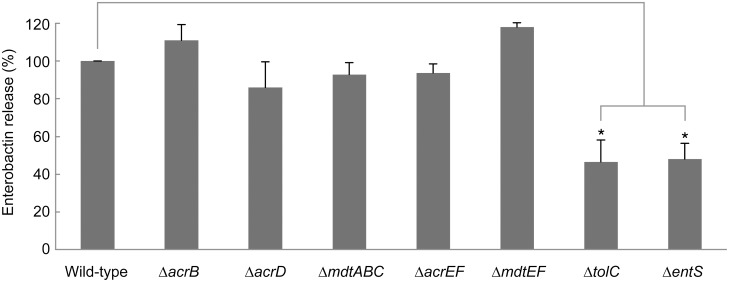
RP HPLC analysis of enterobactin released from deletion mutants of the RND-type efflux system genes. The amounts of enterobactin exported by deletion mutants calculated using each peak area are shown. The peak area corresponding to enterobactin released from the wild-type strain was defined as 100%. The data corresponds to mean values from three independent replicates. The bars indicate standard deviations. Asterisks indicate statistically significant differences (*p*<0.01) according to two-tailed Student’s *t*-tests.

To investigate the role of the TolC-dependent RND-type drug efflux systems on the release of enterobactin from *E. coli*, we performed RP HPLC analysis of the culture supernatants from cultures of *E. coli* MG1655 mutants containing single deletions of the *acrB*, *acrD*, *mdtABC*, *acrEF*, and *mdtEF* genes. We also investigated the effect of the *entS* deletion to confirm that it is involved in enterobactin export [28] Although the amounts of enterobactin released from Δ*tolC* and Δ*entS* were significantly lower than that from the wild-type strain, there was no difference among the Δ*acrB*, Δ*acrD*, Δ*mdtABC*, Δ*acrEF*, Δ*mdtEF*, and wild-type strains (Figure 2). These results are in agreement with a previous report [30].

### AcrB, AcrD, and MdtABC drug efflux systems are required for enterobactin export

**Figure 3:**
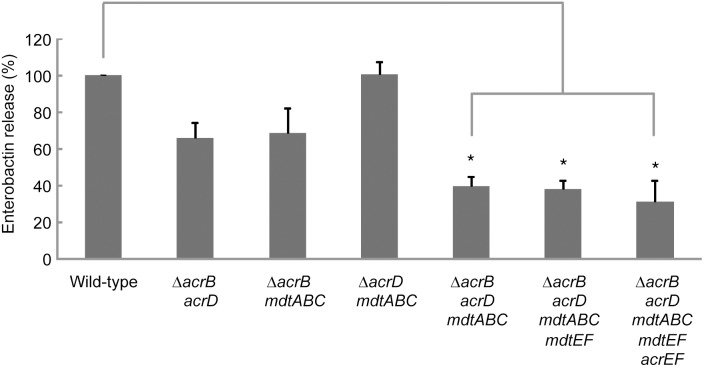
Requirement of AcrB, AcrD, and MdtABC drug efflux systems for enterobactin export. Enterobactin was prepared from the supernatants of cultures of each multiple RND transporter mutant and analyzed by RP HPLC. The amount of enterobactin exported by each strain, calculated using each peak area, is shown. The amount of enterobactin released from the wild-type strain was defined as 100%. The data correspond to mean values from three independent replicates. The bars indicate standard deviations. Asterisks indicate statistically significant differences (*p*<0.01) according to the two-tailed Student’s *t*-tests.

Because individual deletions of the *acrB*, *acrD*, *mdtABC*, *acrEF*, or *mdtEF* gene did not affect enterobactin release from *E. coli*, we hypothesized that multiple efflux systems may be involved in ecterobactin export. To investigate this possibility, we performed analyses using multiple deletion mutants. The growth rates of the double mutants Δ*acrB acrD,* Δ*acrB mdtABC*, and Δ*acrD mdtABC* were almost the same as that of the wild-type strain. The mutants Δ*acrB acrD mdtABC*, Δ*acrB acrD mdtABC mdtEF*, and Δ*acrB acrD mdtABC mdtEF acrEF* grew slightly slower than the wild-type for the initial 3 h after inoculation; however. they grew to the same level as the wild-type after 13 h, when the samples were collected to measure enterobactin release. The double mutants Δ*acrB acrD* and Δ*acrB mdtABC* exported enterobactin to levels 66% and 69% of the level exported by wild type, respectively (Figure 3). Furthermore, deletion of *acrB*, *acrD*, and *mdtABC* also significantly decreased enterobactin export, to only 40% of the level exported by wild type. In contrast, the double mutant Δ*acrD mdtABC* did not alter the ability of the wild-type to export enterobactin. Stepwise deletion of the *mdtEF* and *acrEF* genes from the Δ*acrB acrD mdtABC* mutant did not affect its ability to excrete enterobactin (Figure 3). These data indicate that, among the five RND transporters of *E. coli,* only AcrB, AcrD, and MdtABC play a role in the excretion of enterobactin.

### AcrB, AcrD, and MdtABC are involved in enterobactin export

**Figure 4:**
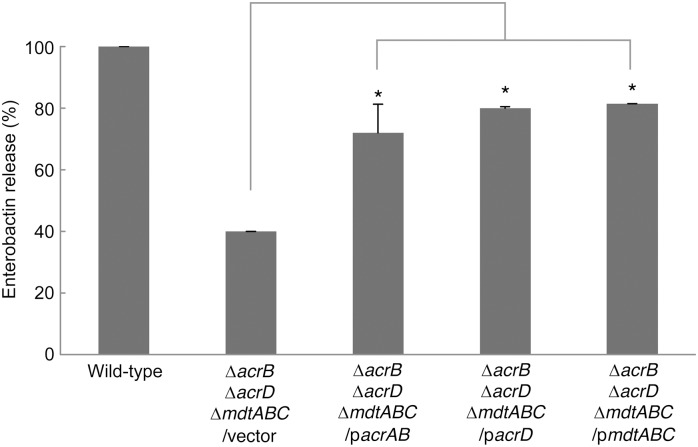
Complementation of enterobactin release from the Δ*acrB acrD mdtABC* mutant using plasmids carrying *acrB*, *acrD*, or *mdtABC* genes. The amounts of enterobactin exported by deletion mutants, calculated using peak areas, are shown. The amount of enterobactin released from the wild-type strain harboring an empty vector was defined as 100%. The data corresponds to mean values from three independent replicates. The bars indicate standard deviations. Asterisks indicate statistically significant differences (*p*<0.01) determined using the two-tailed Student’s *t*-tests.

To confirm that AcrB, AcrD, and MdtABC are involved in enterobactin export, we performed a complementation analysis. The *acrB*, *acrD* and *mdtABC* genes were each cloned into the vector pTrc99A, and the resulting plasmids were used to investigate their ability to complement the enterobactin export defective phenotype of the Δ*acrB acrD mdtABC* mutant. All three plasmids increased enterobactin excretion from the Δ*acrB acrD mdtABC* mutant. The amounts of enterobactin released from the strains complemented with these plasmids were 72%, 80%, and 81% of the level released by the wild-type strain, respectively, which is nearly two-fold greater than that of the Δ*acrB acrD mdtABC* mutant harboring an empty control vector (Figure 4). These data indicates that AcrB AcrD, and MdtABC are involved in enterobactin export in *E. coli*.

## Discussion

In this study, we examined the involvement of the RND transporters in enterobactin export and the results indicate that AcrB, AcrD, and MdtABC are required for the secretion of enterobactin. The iron-chelating compound enterobactin is synthesized in the cytoplasm and exported to the growth medium to acquire iron. The MF-type transporter EntS was previously shown to be involved in enterobactin transport across the cytoplasmic membrane [28]. It has been believed that transporters in addition to EntS are involved in enterobactin export. Outer membrane channel TolC was proposed as a candidate for enterobactin export because of its wide substrate specificity [39] and ability to cooperate with multiple systems, including MF- and RND-type transporters [40], [41]. Another study demonstrated that deletion of *tolC* leads to the abolishment of enterobactin export and suggested that TolC is involved in enterobactin export from the periplasm to the growth medium [30]. In our study, deletion of *tolC* resulted in a decrease in enterobactin release, but it did not result in a complete loss of the ability to excrete enterobactin; the *tolC* deletion mutant could still export enterobactin to some extent. We believed that this resulted, in part, from differences in strain background. We used wild-type MG1655 as the background strain, but Δ*fur* was used in a previous study [30]. Fur encodes the global regulator of iron homeostasis and deletion of *fur* results in constitutive production of enterobactin [42]. Because deletion of *fur* can affect enterobactin production and export, a *tolC* deletion may show a greater effect in this genetic background.

**Figure 5:**
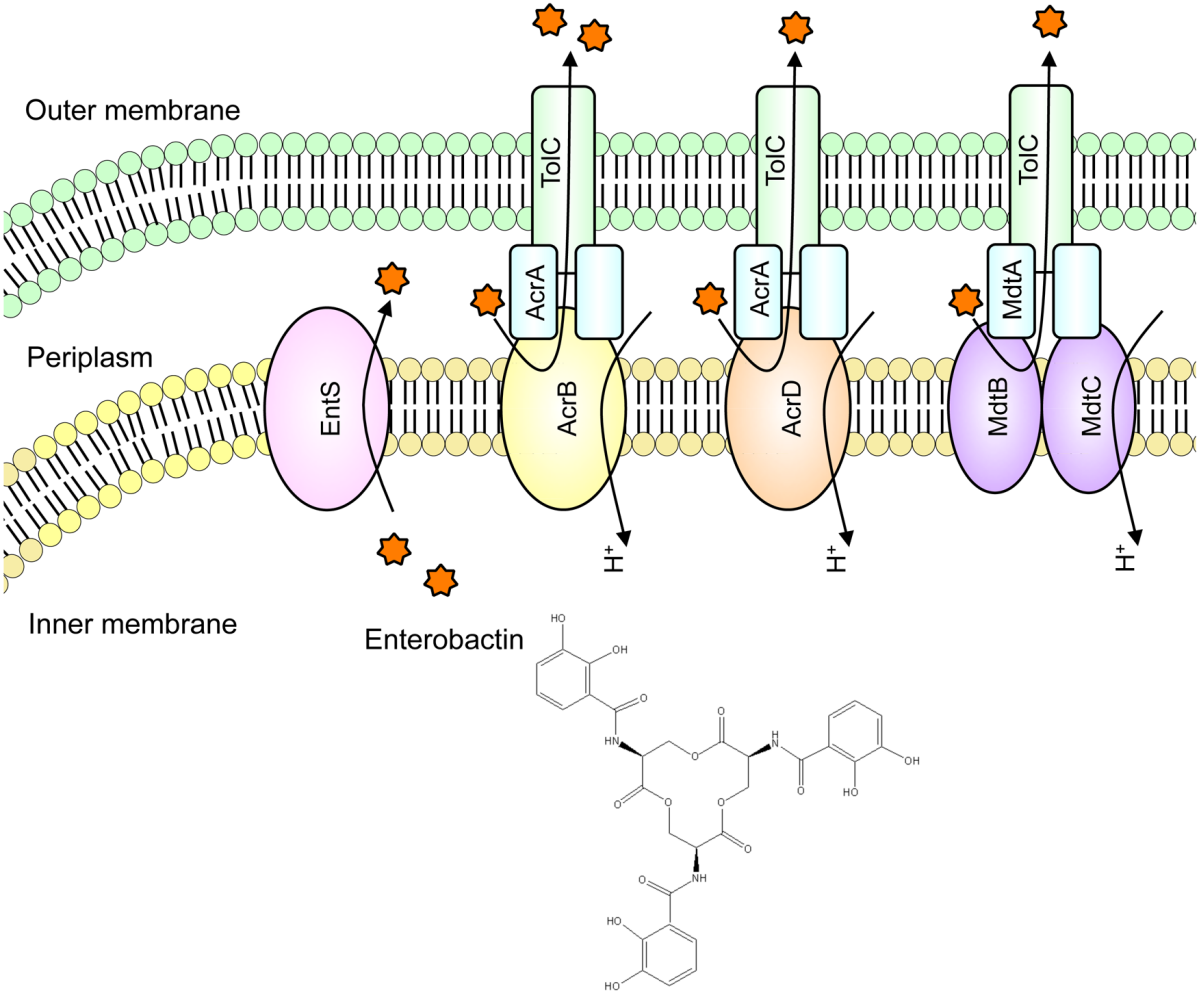
Proposed model of enterobactin export in *E. coli.* Enterobactin is synthesized in the cytoplasm and exported to the periplasm by EntS. The RND transporters AcrB, AcrD, and MdtABC capture enterobactin in the periplasm and then export it to the growth medium throughout the outer membrane channel TolC.

Individual deletions of *acrB*, *acrD*, and *mdtABC* did not affect the ability of *E. coli* cells to excrete enterobactin, whereas a triple deletion of these genes resulted in a significant decrease in enterobactin export. These three genes may not be unique in their ability to mediate enterobactin excretion, but AcrB, AcrD, and MdtABC coordinately play a role in enterobactin export from the periplasm to the growth medium in *E. coli* (Figure 5). Considering the results that double mutants Δ*acrB acrD* and Δ*acrB mdtABC* showed a decrease in enterobactin export, whereas Δ*acrD mdtABC* did not change its ability to export enterobactin, we speculate that AcrB plays a more pivotal role than AcrD and MdtABC.

The AcrAB–TolC system is constitutively expressed, but the expression levels of *acrD* and *mdtABC* are quite low under normal conditions [43]. We speculate the *acrD* and *mdtABC* genes may be induced when bacteria require iron to survive. Recently, our study on the identification of negative regulators for *acrD* and *mdtABC* revealed that the expression levels of these genes were affected by Fur (data not shown). A recent study by Ruiz and Levy also showed that inactivation of the enterobactin biosynthetic genes affects the expression level of *acrAB* [44]. These results suggest that multidrug transporters contribute to bacterial iron homeostasis, in addition to their role in multidrug resistance.
